# Annual acknowledgement of manuscript reviewers

**DOI:** 10.1186/1475-2859-12-11

**Published:** 2013-02-19

**Authors:** Antonio Villaverde

**Affiliations:** 1 Institut de Biotecnologia i de Biomedicina, Universitat Autònoma de Barcelona, Bellaterra, Barcelona, 08193, Spain; 2 Department de Genètica i de Microbiologia, Universitat Autònoma de Barcelona, Bellaterra, Barcelona, 08193, Spain; 3 CIBER de Bioingeniería, Biomateriales y Nanomedicina (CIBER-BBN), Bellaterra, Barcelona, 08193, Spain

## Abstract

**Contributing reviewers:**

The Editors of Microbial Cell Factories would like to thank all our Reviewers who have contributed to the journal in Volume 11 (2012).

## 

Marcio Acencio

Brazil

Pratul Agarwal

United States of America

Rinji Akada

Japan

Pablo Alvira

Spain

Alberto Amaretti

Italy

Ricardo Amils

Spain

David Archer

United Kingdom

Shota Atsumi

United States of America

Bernhard Auer

Austria

Vasco Azevedo

Brazil

Fernanda Badotti

Brazil

Antonio Ballesteros

Spain

Paolo Barghini

Italy

Abolfazl Barzegari

Iran

Karl Bayer

Austria

Dennis Bazylinski

United States of America

Athanasios Beopoulos

France

Rita Bernhardt

Germany

Jean-Guy Berrin

France

Magnus Bertilsson

Sweden

Katja Bettenbrock

Germany

Jean-michel Betton

France

Sanjoy K. Bhattacharya

United States of America

Roslyn Bill

United Kingdom

Josep Biosca

Spain

Louise Elizabeth Bird

United Kingdom

Lars Mathias Blank

Germany

Marc Blondel

France

Bruno Blondin

France

Michael Boelker

Germany

Eckhard Boles

Germany

Daniel Bond

United States of America

Ian Booth

United Kingdom

Irina Borodina

Denmark

Daniel Bracewell

United Kingdom

Ulrich Brandt

Germany

Paola Branduardi

Italy

Michael Breitenbach

Austria

Erhard Bremer

Germany

Elisabetta Brenna

Italy

Hans Brink

Denmark

Melanie Brocker

Germany

Peter Bron

Netherlands

Per Bruheim

Norway

Stevens Brumbley

United States of America

Jochen Büchs

Germany

Mark Caddick

United Kingdom

Lei Cai

China

Pinar Calik

Turkey

M. Angeles Calvo

Spain

Swaranjit Singh Cameotra

India

George Carman

United States of America

James Carothers

United States of America

Francisco Carrau

Uruguay

Margarida Casal

Portugal

Hyung Joon Cha

Korea, South

Paul-Philippe Champagne

Canada

Jo-Shu Chang

Taiwan

Joe Chappell

United States of America

Guo-Qiang Chen

China

Rachel Chen

United States of America

Wilfred Chen

United States of America

Jong Hyun Choi

Korea, South

Ah-Leum Chung

China

Victor Cifuentes

Chile

Pieternel Claasse

Netherlands

Fabian Commichau

Germany

José Luis Corchero

Spain

Ricardo Cordero-Otero

Spain

Naima Gisela Cortes-Perez

France

Paolo costantino

Italy

Peggy Cotter

United States of America

Don Cowan

South Africa

Marc Creus

Switzerland

Roger Cripps

United Kingdom

Simon Cutting

United Kingdom

Robert Czajkowski

Netherlands

Jiri Damborsky

Czech Republic

Nancy DaSilva

United States of America

Laura Dato

Italy

Michael Dauner

United States of America

Maurilio de Felice

Italy

Jan-Willem de Gier

Sweden

Victor de Lorenzo

Spain

Marizela Delic

Austria

Frank Delvigne

Belgium

Katerina Demnerova

Czech Republic

Riaan Den Haan

South Africa

Sylvie Dequin

France

Mario Díaz

Spain

Juana Diez

Spain

Neal Dittmer

United States of America

Bien Cuong Do

Viet Nam

Lucilia Domingues

Portugal

Stefano Donadio

Italy

Adam Driks

United States of America

Mary Dunlop

United States of America

Mark Eiteman

United States of America

Giovanni Emiliani

Italy

Sven-Olof Enfors

Sweden

Rawi Fakhrullin

Russian Federation

Fabio Fava

Italy

Isabel Ferreira

Portugal

Pau Ferrer

Spain

Neus Ferrer-Miralles

Spain

Andrzej Fertala

United States of America

Andre Fleissner

Germany

Ann Flower

United States of America

Debora Foguel

Brazil

Luis Fonseca

Portugal

Jean Marie Francois

France

Mary Ann Franden

United States of America

Paul Fraser

United Kingdom

Annie Frelet-Barrand

France

Roland Freudl

Germany

Karl Friehs

Germany

Michael Gänzle

Canada

Elena Garcia-Fruitos

Spain

Brigitte Gasser

Austria

George Georgiou

United States of America

Paola Giardina

Italy

Anton Glieder

Austria

Andreas Gombert

Brazil

Ramon Gonzalez

United States of America

Jesus Gonzalez-Lopez

Spain

M. Isabel González-Siso

Spain

Marie-F Gorwa-Grauslund

Sweden

Corinne Grangette

France

Víctor Gabriel Guadalupe Medina

Netherlands

Meijin Guo

China

Daniela Gurpilhares

Brazil

Lee Haines

United Kingdom

Wataru Hakamata

Japan

Patrick Hallenbeck

Canada

Norbert Hampp

Germany

Mee-Jung Han

Korea, South

Nam Soo Han

Korea, South

Piyanun Harnpicharnchai

Thailand

Colin Harwood

United Kingdom

Alexander Haslberger

Austria

Tomohisa Hasunuma

Japan

Jun He

China

Michael Hecker

Germany

Elmar Heinzle

Germany

Klaas Jan Hellingwerf

Netherlands

Thomas Herfellner

Germany

Baharak Hosseinkhani

Belgium

Gerardo Huerta-Beristain

Mexico

David Hulmes

France

William J (Jim) Hunter

United States of America

Michael Hust

Germany

Zoya Ignatova

Germany

Marja Ilmen

Finland

Mehmet Inan

Turkey

Lonnie Ingram

United States of America

Masayuki Inui

Japan

Francoise Jacob-Dubuisson

France

Sylwia Jafra

Poland

Laura Jarboe

United States of America

Jolanta Jaroszuk-Ściseł

Poland

Thomas Jeffries

United States of America

Zhengbing Jiang

China

Yong-Su Jin

United States of America

Leif J Jonsson

Sweden

Christoph Kaleta

Germany

Héla Kallel

Tunisia

Rainer Kalscheuer

Germany

Katy Kao

United States of America

Michihiko Kataoka

Japan

Margaet Katz

Australia

Jay D Keasling

United States of America

Nancy Keller

United States of America

Frank Kensy

Germany

Donghak Kim

Korea, South

Hak-Sung Kim

Korea, South

Jan Kok

Netherlands

Martin Koller

Austria

Robert Kolodziejczyk

Finland

Daisuke Koma

Japan

Akihiko Kondo

Japan

Nadia Krieger

Brazil

Otini Kroukamp

Canada

Christian P. Kubicek

Austria

Oscar Kuipers

Netherlands

Jean Labarre

France

William Laing

New Zealand

Christine Lang

Germany

Kobkul Laoteng

Thailand

Alvaro R. Lara

Mexico

Gen Larsson

Sweden

Sarah Lebeer

Belgium

Byong Lee

Canada

Dong-Woo Lee

Korea, South

Pyung Cheon Lee

Korea, South

Anne L’Hernault

United Kingdom

Guojian Liao

China

James Liao

United States of America

Yonghua Li-Beisson

France

Savitree Limtong

Thailand

Zhanglin Lin

China

Mirjana Liovic

Slovenia

Boris Litsanov

Switzerland

Shuang-Jiang Liu

China

Małgorzata Łobocka

Poland

Anibal Lodeiro

Argentina

Elisabeth Lojou

France

Ana Maria Lopez-Contreras

Netherlands

Josep Lopez-Santin

Spain

Marina Lotti

Italy

Ming-Yeou Lung

Taiwan

Hongwu Ma

United Kingdom

Astrid Rosa Mach-Aigner

Austria

Juergen Mairhofer

Austria

Daniel Manter

United States of America

Ian Marison

Ireland

Juan-Francisco Martin

Spain

Alfredo Martinez

Mexico

Isabelle Martin-Verstraete

France

Fumio Matsuda

Japan

Diethard Mattanovich

Austria

Filip Matthijssens

Belgium

Julie Maupin-Furlow

United States of America

Jérôme Maury

Denmark

Lotfi Mellouli

Tunisia

Guido Melzer

Germany

Hugo G Menzella

Argentina

Joris Messens

Belgium

Isabelle Meynial-Salles

France

Andrei Miasnikov

United States of America

Jonathan Mielenz

United States of America

Mario Mirisola

Italy

Ralf Moeller

Germany

Axel Mogk

Germany

Andrea Moglia

Italy

Francesco Molinari

Italy

Diego Mora

Italy

Antonio Moreira

United States of America

John Morrissey

Ireland

Michael Mourez

Canada

Krishna Jyoti Mukherjee

India

Rolf Müller

Germany

Jozef Nahalka

Slovakia

Tomoyuki Nakagawa

Japan

Carmen Mihaela Neculita

Canada

Darren Nesbeth

United Kingdom

Peter Neubauer

Germany

Helena Nevalainen

Australia

Bernd Nidetzky

Austria

Jens Nielsen

Sweden

Stephan Noack

Germany

Philippe Noirot

France

Eugenio Notomista

Italy

Naotake Ogasawara

Japan

Yasuo Ohnishi

Japan

Marco Oldiges

Germany

Sahlan Ozturk

Turkey

Seung Pil Pack

Korea, South

Laura Palomares

Mexico

Christos Panagiotidis

Greece

Gianni Panagiotou

Denmark

Amulya Panda

India

Francesca Paradisi

Ireland

Tae Jung Park

Korea, South

Victor Parro

Spain

Hugo Peluffo

Uruguay

Kugen Permaul

South Africa

Spela Peternel

Slovenia

Maike Petersen

Germany

Maria Julia Pettinari

Argentina

Brian Pfleger

United States of America

Vincent Phalip

France

Je-Nie Phue

United States of America

Harald Pichler

Austria

Markus Piotrowski

Germany

Maria de Lourdes TM Polizeli

Brazil

Isabelle Poquet

France

Danilo Porro

Italy

Gyorgy Posfai

Hungary

Anna Maria Puglia

Italy

Pablo Pulido

Spain

Xinghui Qiu

China

Odilia Queiros

Portugal

Noura Raddadi

Italy

Jeyaprakash Rajendhran

India

Palanisamy Rani

India

Mala Rao

India

Kenneth Reardon

United States of America

Gregor Reid

Canada

David Resina

Spain

Moez Rhimi

France

Peter Richard

Finland

Ursula Rinas

Germany

Robera Rizzo

Italy

Karen Robins

Switzerland

Anne Robinson

United States of America

Isabel Rocha

Portugal

Jesus Rodriguez-Diaz

Spain

Susana Romero-Garcia

Mexico

Eliora Ron

Israel

German Rosano

Argentina

Franca Rossi

Italy

Neil Rowan

Ireland

Harald Ruijssenaars

Netherlands

Wael Sabra

Germany

Kazuo Sakka

Japan

Laura Salusjärvi

Finland

Sergio Sanchez

Mexico

Suvi Santala

Finland

Eduardo Santero

Spain

Moises Santillan

Mexico

Michael Sauer

Austria

Uwe Sauer

Switzerland

Olga Sayanova

United Kingdom

Joseph Schacherer

France

Andreas Schmid

Germany

Ghislain Schyns

Switzerland

Maria Mercedes Segura

Spain

Jin-Ho Seo

Korea, South

Enrique Seoane-Vazquez

United States of America

Alain L. Servin

France

Luisa Seuanes Serafim

Portugal

Fergus Shanahan

Ireland

Yixin Shi

United States of America

Joseph Shiloach

United States of America

Andriy Sibirny

Ukraine

Volker Sieber

Germany

Jose-Vicente Sinisterra

Spain

Eva Sinner

Austria

Christopher Skory

United States of America

Nicholas Smirnoff

United Kingdom

Stephen Smith

Ireland

Danièle Sohier

France

Lijiang Song

United Kingdom

Shuishan Song

China

Bernhard Sonnleitner

Switzerland

Fani Sousa

Portugal

Oliver Spadiut

Austria

Georg Sprenger

Germany

Andrea Squartini

Italy

Boris Stambuk

Brazil

Alexander Steinbuchel

Germany

Gerald Striedner

Austria

Kumar Sudesh

Malaysia

Michael Sullivan

United States of America

Agnieszka Szalewska-Palasz

Poland

Seiichi Taguchi

Japan

Ralf Takors

Germany

Patricia Taillandier

France

Yinjie Tang

United States of America

Andreas Tauch

Germany

Michael Ter-Avanesyan

Russian Federation

Alessandro Terrinoni

Italy

Johan Thevelein

Belgium

Linda Thöny-Meyer

Switzerland

Chaoguang Tian

China

Mervi Toivari

Finland

Santiago Torres-Martínez

Spain

Cong Trinh

United States of America

Kouhei Tsumoto

Japan

Vlada Urlacher

Germany

Marina Vai

Italy

Sabeel P Valappil

United Kingdom

Francisco Valero

Spain

Svein Valla

Norway

Fernando Valle

United States of America

Bert van den Berg

United States of America

Menno van der Voort

Netherlands

Jan Maarten van Dijl

Netherlands

Jan van Doorsselaere

Belgium

Ed van Niel

Sweden

Willem H van Zyl

South Africa

Giovanna Cristina Varese

Italy

Dimitris Vayenas

Greece

Douwe Veltman

United Kingdom

Mauritz Venter

South Africa

Andre Vermeglio

France

Karen Visick

United States of America

Birgit Voigt

Germany

Wanwipa Vongsangnak

China

Gerard Wall

Ireland

Jens Walter

United States of America

David Waugh

United States of America

Grzegorz Wegrzyn

Poland

Kira Weissman

France

Volker Wendisch

Germany

Thomas West

United States of America

Wolfgang Wiechert

Germany

Nick Wierckx

Germany

Christoph Wittmann

Germany

Roland Wohlgemuth

Switzerland

Jing Wu

China

Xin-Hui Xing

Japan

Zongming Xiu

United States of America

Yajun Yan

United States of America

Tae Hoon Yang

United States of America

Zhen Yang

China

Bin Yao

China

Chuan Mei Yeh

Taiwan

Alexei Yeliseev

United States of America

Chul-Ho Yun

Korea, South

Hiroya Yurimoto

Japan

Rintze Zelle

United States of America

Kechun Zhang

United States of America

Shengde Zhou

United States of America

Wen Zhou

United States of America

Georgia Zoumpopoulou

Greece

